# Genome Survey and Transcriptome Analysis on Mycelia and Primordia of *Agaricus blazei*

**DOI:** 10.1155/2020/1824183

**Published:** 2020-01-13

**Authors:** Yuan-Ping Lu, Jian-Hua Liao, Zhong-Jie Guo, Zhi-Xin Cai, Mei-Yuan Chen

**Affiliations:** Institute of Edible Fungi, Fujian Academy of Agricultural Sciences, Fuzhou 350014, Fujian Province, China

## Abstract

*Agaricus blazei*, a type of edible straw-rotting mushroom with somewhat sweet taste and fragrance of almonds, has attracted considerable scientific and practical attention. High-throughput Illumina PE150 and PacBio RSII platform were employed to generate a genomic sequence. De novo assembly generated 36 contigs with 38,686,133 bp in size, containing 10,119 putative predicted genes. Additionally, we also studied transcriptional regulation of the mycelia and the primordia for exploration of genes involved in fruiting body formation. Expression profiling analysis revealed that 2,164 genes were upregulated in mycelia and 1,557 in primordia. Functional enrichment showed that differentially expressed genes associated with response to stress, ribosome biogenesis, arginine biosynthesis, and steroid biosynthesis pathway were more active in fruiting body. The genome and transcriptome analysis of *A. blazei* provide valuable sequence resources and contribute to our understanding of genes related to the biosynthesis pathway of polysaccharide and benzaldehyde, as well as the fruiting body formation.

## 1. Introduction

Mushrooms (mainly the fruiting body) have been used for consumption as a product by humans since ancient times due to their delicacy and nutritional values, as well as their medicinal properties [1, 2]. *Agaricus blazei* Murrill, an edible mushroom originating from Brazil, is commonly known as “Cogumelo do Sol” in Brazil, “Himematsutake” in Japan, and “Ji Song Rong” in China [3, 4]. It serves as one of the utmost valuable edible and culinary-medicinal Royal Sun mushroom species. Also, it has been widely consumed today in several Oriental countries and studied for its high nutritional properties and pharmacological effects, such as antitumor activity [5], antiviral effect [6], antidiabetic potential [7], and antiapoptotic role [8]. Nowadays, *A. blazei* has received great scientific and practical interest. A series of bioactive compounds, for instance polysaccharides [9, 10], lectin [11, 12], and ergosterol [13, 14], have been discovered in *A. blazei*. Moreover, several volatile flavor ingredients have been identified in its fruiting body and mycelia [15]. However, very few studies focus on those compounds' biosynthesis pathways and the fruiting body formation in *A. blazei*.

Recently, several studies reported genome or transcriptome landscape of mushrooms, such as *Agaricus bisporus* [16], *Volvariella volvacea* [17, 18], *Flammulina velutipes* [19], and *Auricularia heimuer* [20]. The availability of genomes has been a benefit to researchers for identification of CAZymes, mating-type loci, *β*-glucan synthase genes, and secondary metabolite genes [17, 18, 20]. Additionally, transcriptome or comparative expression profiles from different developmental stages have facilitated the discovery of genes involved in fruiting body formation [21–23]. The shift from vegetative mycelia to primordia has been seen as one of the most complex and critical developmental processes in mushrooms [24]. Understanding the molecular mechanism regulating fruiting body formation will contribute to the improvement of commercial mushroom production, with consequent economic benefits.

In this study, we presented the genome of *A. blazei* to understand the genomic structure and its gene content. Additionally, we also performed the transcriptome analysis of mycelia and primordia to study the expression difference between the two stages. The transcriptome analysis will enable us to identify genes regulating mycelia growth and fruiting body formation. Our genomic and transcriptomic data will provide important information platform for the further investigation of this species.

## 2. Materials and Methods

### 2.1. Strains and Culture Condition

*A. blazei* strain JA, the main cultivar in several provinces in China (eg., Fujian, Yunnan, Jiangxi, Guangxi, Henan, and Sichuan province), was provided by Fujian Academy of Agricultural Sciences. To prepare samples for RNA sequencing, strain JA was cultured on a compost purchased from a company producing button mushroom (Jinming Food Co., Ltd, Fujian, China), and the main components of the compost are rice straw, bagasse, cow manure, and gypsum. Samples of mycelia (20 days after casing) and primordia (24 days after casing, equivalent to the pinhead stage in *A. bisporus* whose velum not differentiated according to Hammond and Nichols [25]) were harvested and immediately frozen in liquid nitrogen. For the whole genome sequencing, *A. blazei* sterile monospore strain JA-15036, germinated from one of the spores of dikaryotic strain JA, was incubated in liquid potato dextrose broth at 24°C for 20 days.

### 2.2. DNA Sequencing and Genome Assembly

Genomic DNA was extracted with the SDS (sodium dodecyl sulfate) method and sequenced by Novogene Biotech AG (Beijing, China) on the Illumina PE150 and PacBio RSII platform. Paired-end reads were constructed after sequencing of a 350 bp insert library using an IlluminaHiseq X system. For PacBio RSII platform, a 20 kb library was generated and sequenced.

Prior to assembly, reads of low quality were filtered by the following steps. For preprocessing the raw data of the 350 bp library, reads with a certain proportion of low-quality (read quality ≤35) bases (40% as default, parameter setting at 40 bp) were removed from raw data; reads containing a certain percentage of Ns' base or low complexity reads (parameter setting at 10 bp) were filtered out; adapter contaminations (15 bp overlap between reads and adapter) and duplication contaminations were filtered out. Reads of PacBio RSII were filtered using SMRT Link v5.0.1 software with default parameters [26, 27], after which PacBio data were de novo assembled using SMRT Link v5.0.1 software into contigs with default parameters. And then, the Arrow algorithm in SMRT Link software was applied to polish the assembled contigs. We assessed the completeness of genome assembly using BUSCO v3.0.2 (Benchmarking Universal Single-Copy Orthologs) software (using “-m genome”) [28]. The lineage dataset of BUSCO is fungi_odb9 (creation date: 2016-02-13, number of species: 85, and number of BUSCOs: 290).

### 2.3. Genome Annotation and Identification of Carbohydrate-Active Enzymes

Protein-coding genes were predicted using Augustus (version 2.7) [29]. All of these genes were annotated by analysis of the corresponding amino acid sequences with the GO (Gene Ontology database), KEGG (Kyoto Encyclopedia of Genes and Genomes), KOG, NR (nonredundant), and SwissProt protein database using Blastp (*E* value ≤ 1*e*^−5^).

We employed the tRNAscan-SE (Version 1.3.1) [30] and rRNAmmer [31] to analysis transfer RNA (tRNA) and ribosome RNA (rRNA) genes, respectively. sRNA, snRNA, and miRNA were predicted by BLAST against the Rfam database [32, 33]. The interspersed repetitive sequences were predicted using the RepeatMasker (http://www.repeatmasker.org/), and the tandem repeats [34] were analyzed using the TRF (tandem repeats finder) [35].

Annotations of carbohydrate-active enzymes (CAZymes) in the *A. blazei* genome were performed by BLASTP search of CAZymes database at http://www.cazy.org/ (*e* value ≤ 1*e* − 5; the covered fraction ratio ≥40%; and minimal alignment length percentage ≥40%).

### 2.4. Transcriptome Analysis during Fruiting Bodies Formation

Total RNA of each sample from *A. blazei* heterokaryon strain JA was sequenced on the Illumina HiSeq X Ten Station and Illumina PE150 platform at Novogene Biotech. In order to ensure the accuracy of the further analysis, clean data (clean reads) were generated by trimming of reads containing adapter sequences, reads with unknown sequences Ns, and low quality reads which contained bases with *Q*_phred_ ≤ 20, and the percentage of these bases was more than 50% of a read. And then, RNA-seq clean reads from each sample were separately aligned to the reference genome using software Hisat2v2.0.5 [36], and novel genes were predicted by StringTie [37].

For obtaining the differentially expressed genes, feature Countsv1.5.0-p3 software was adopted to count the numbers of reads mapped to each gene [38, 39], after which the two samples (each with three replicates) were analyzed using DESeq2R package (1.16.1) [40], with the cutoff threshold, adjusted *P* value <0.05, and |log2Ratio| ≥ 1. For function enrichment analysis, differential expression genes (DEGs) were aligned in the GO database. The pathway enrichment analysis was implemented using the KEGG database. In both analyses, cluster Profiler Rpackage [41] was applied and the correct *P* value less than 0.05 was regarded as the threshold.

### 2.5. Quantitative Real-Time PCR (qRT-PCR) Validation

To assess the reliability of the RNA sequencing-based approach in identifying DGEs, qRT-PCR was employed to detect gene transcript patterns. One microgram of total RNA from the mycelia and primordia was adopted to synthesize cDNA using TransScript All-in-One First-Strand cDNA Sythesis Super Mix for qPCR kit (TransGen Biotech, Beijing) in accordance with the manufacturer's protocol. The primers ([Supplementary-material supplementary-material-1]) used for quantitative real-time PCR (qRT-PCR) analysis were designed with primer premier 5.0 [42]. Twenty microliters of qRT-PCR reaction mixtures were prepared according to the manufacturer's instructions using TransStart TOP Green qPCR kit (TransGen Biotech, Beijing). The cycling parameters were as follows: 94°C for 30 s followed by 30 cycles of 94°C for 5 s and 60°C for 30 s. Three independent biological replicates were carried out for each gene. The glyceraldehyde-3-phosphate dehydrogenase gene (*GAPDH*) and *α-tubulin* severed as an internal control gene, and the 2^−ΔΔ*ct*^ method [43] was applied to the calculated gene expression.

## 3. Result and Discussion

### 3.1. General Features of the Genome

We constructed genome sequence data for *A. blazei* homokaraytic strain JA-15036 by combining a high-throughput Illimina Hiseq PE150 with a PacBio RSII long-read sequencing platform (Tables 1 and 2). A 38,686,133 bp genome sequence was generated by assembling PacBio clean data. This genome assembly consisted of 36 contigs with an N50 of 1,826,870 base pairs (Table 3). The *A. blazei* genome is similar in size to the genomes of several other fruiting body formation fungi from the other Agaricales, including *Schizophyllum commune* (38.5 Mb) [44], *V. volvacea* (37.2 Mb) [17], and *Coprinopsis cinerea* (37 Mb) [45], but larger than that of *Pleurotus ostreatus* (34.9 Mb) [46] and *A. bisporus* (30.2 Mb) [16].

The completeness of genome was assessed using BUSCO software, and the result (Table 4) suggested a well-completed annotation set, with 93.7% of the Fungi BUSCOs within the RefSeq annotation set and 2.1% of fragmented.

**Figure 1:**
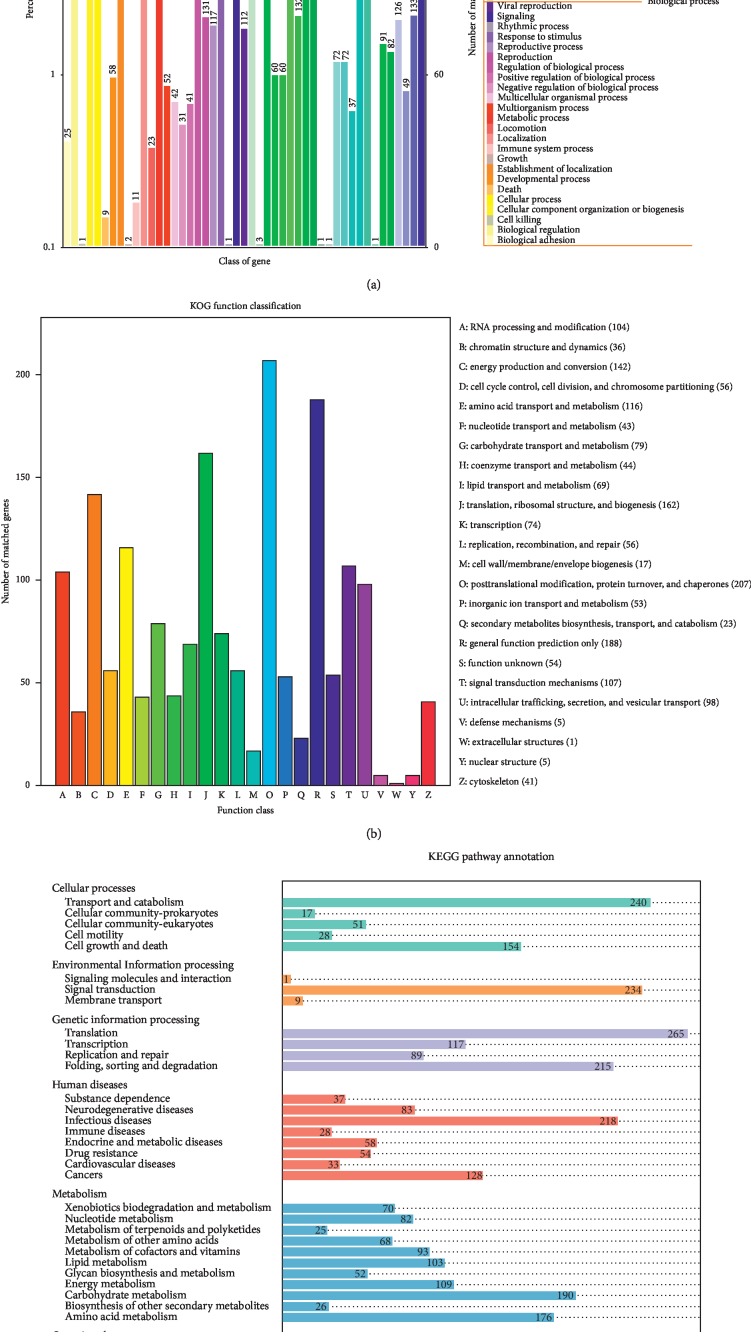
Annotation of *A. blazei* genome with GO, KOG, and KEGG. (a) *A. blazei* gene GO analysis; (b) KOG function classification of *A. blazei*; and (c) *A. blazei* gene KEGG pathway classification.

A total of 10,119 genes ([Supplementary-material supplementary-material-1]) were predicted by Augustus (version 2.7). Approximately 9,174 (90.7%) genes were annotated in similarity search with GO, KEGG, KOG, NR, CAZY, Pfam, and Swiss-Prot databases (Table 3). The remaining 945 (9.3%) predicted genes without apparent homolog to the currently available sequences and protein domains were found, and these genes were presumed to be the specific genes in *A. blazei* genome. Among the 10,119 genes, 8,694 (85.9%) genes encoded proteins with homologous sequences in the NCBI NR databases, and 8,538 predicted proteins accounting for 84.4% of the entire genome were mappable through the KEGG pathway database (Table 3). The KOG analysis indicated that 1,620 genes were assigned to different KOG categories (Figure 1(a)), and GO analysis revealed 6,064 proteins into 3 different GO terms (biological process, cellular component, and molecular function) (Figure 1(b)). In addition, KEGG analysis assigned 1,768 predicted proteins involved in different pathways (Figure 1(c)).

The total length of repeat sequences in the ∼38.7 Mb assembled genome of *A. blazei* was 1,961,315 bp, accounting for 5.0698%. Of the repeat elements, tandem repeat sequences account for 1.1761% and interspersed nuclear elements were 3.8937% (long terminal repeats (LTRs), 3.5795; DNA, 0.2483%; short interspersed nucleotide elements (SINEs), 0.0027%; long interspersed nucleotide elements (LINEs). 0.0589%; rolling circle (RC), 0.0138%; and unclassified, 0.0051%) of the assembled genome.

### 3.2. The CAZymes in *A. blazei* Genome

CAZymes are associated with the degradation of plant cell wall polysaccharides and have an important role in the processes of substrate degradation [17]. Identification of CAZymes will facilitate the further exploration of mechanisms of plant polysaccharide hydrolysis. In *A. blazi* genome, a total of 279 candidate genes encoding CAZymes were identified, including 135 glycoside hydrolases (GHs), 36 glycosyl transferases (GTs), 5 polysaccharide lyases (PLs), 14 carbohydrate esterases (CEs), 74 auxiliary activities (AAs), and 39 carbohydrate-binding modules (CBMs) ([Supplementary-material supplementary-material-1]). The number of GHs family, involved in the cell wall polysaccharides decomposition [47], was significantly larger than that of GTs, which might be because of its lifestyle in which cell wall polysaccharides, such as lignin and cellulose, provide nutrition for its survival.

### 3.3. Identification of Genes Involved in *β*-1,3-Glucan and UDP-Glucose Biosynthesis

Polysaccharides have been considered to be the main component of *A. blazei* for antitumor [4, 48–50]. Among the polysaccharides in the fungal cell wall, water soluble *β*-1,3-glucan with anticancer activity has been widely applied for pharmaceutical purpose [41]. Biosynthesis of *β*-1,3-glucan begins with the formation of a nucleoside diphosphate. Uridinediphosphate glucose (UDP-glucose) acts as a precursor of *β*-1,3-glucan, and its synthesis requires hexokinase or glucokinase, *α*-phosphoglucomutase, and UTP-glucose-1-phosphate uridylyltransferase [20, 51, 52]. Genes encoding these enzymes were identified in *A. blazei* genome ([Supplementary-material supplementary-material-1]).

**Figure 2:**
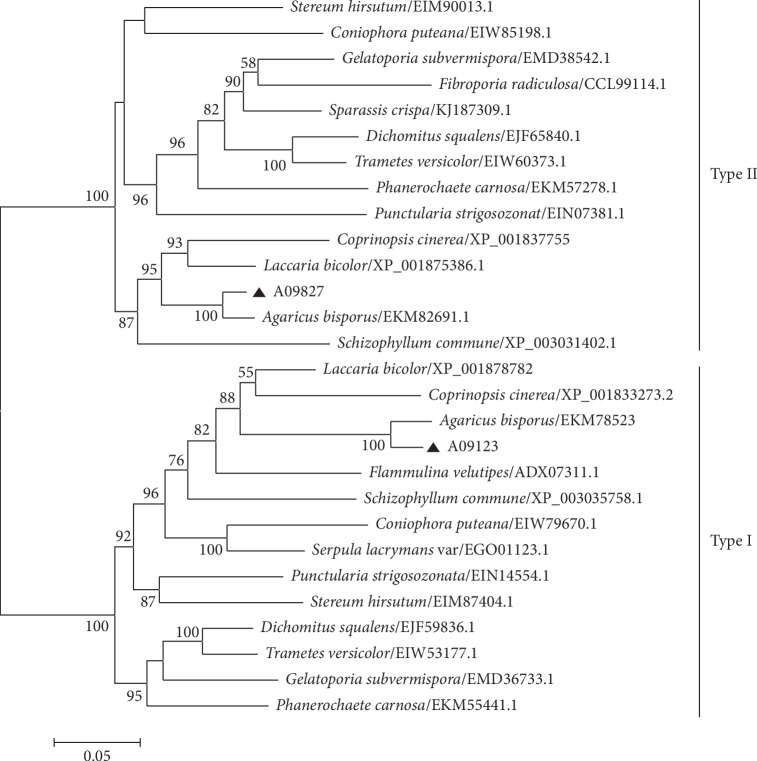
Phylogenetic analysis of *A. blazei β*-1,3-glucan synthases with amino acid sequences of *β*-1,3-glucan synthases identified from different fungi, according to Yang et al. [55]. The construction of phylogenetic tree was implemented on http://www.phylogeny.fr/, as described by Lu et al. [56]. The triangle indicated *β*-1,3-glucan synthases from *A. blazei*.

*A. blazei* also possessed two potential *β*-1,3-glucan synthase genes (A09123 and A09827, named *AbFKS1* and *AbFKS2*, respectively) known to play an important role in *β*-1,3-glucan biosynthesis [52–54]. *AbFKS1* and *AbFKS2* shared high similarity to two A. *bisporusβ*-1,3-glucan synthase genes EKM78523 and EKM82691.1 with the identity of 93% and 90%, respectively, and they were classified into two types (Figure 2). There are significant similarities in domains between AbFKS1 and AbFKS2, where we observed conservation of the catalytic FKS1 domain (*E* value = 1.3*e* − 41 and 1.1*e* − 41, respectively) and *β*-glucan synthase-homologous region (*E* value = 0) using Pfam analysis (http://pfam.xfam.org/search).

Previous studies showed that *Saccharomyces cerevisiae* was found to contain two independent genes *FKS1* and *FKS2* encoding two functionally independent *β*-glucan synthases [54]. FKS1 was primarily expressed in the vegetative growth, whereas FKS2 was expressed under stress conditions (include sporulation and starvation) and mating process [54, 57]. As there is no knowledge about whether the two types of *β*-1,3-glucan synthase will have differences in catalytic activity and function during developmental process in *A. blazai* or not, more experiments (eg., RNAi and overexpression) will be performed to explore their function in our future studies.

### 3.4. Biosynthesis of Benzaldehyde and Benzyl Alcohol in *A. blazei*

The aromatic compounds of edible mushrooms, such as benzyl alcohol, benzaldehyde, benzonitrile, and a phenyl acetic acid-like compound, can whet the appetite. Among the volatile components, benzaldehyde and benzyl alcohol are considered as the main volatile flavor compounds in *A. blazei*. The production of benzaldehyde and benzyl alcohol from phenylalanine has been investigated in various microorganisms [58–62]. In addition, the conversion of phenylalanine to benzaldehyde and benzyl alcohol requires several enzymes. Here, we found 34 enzymes that might be associated with the biosynthesis of benzaldehyde and benzyl alcohol in *A. blazei* genome ([Supplementary-material supplementary-material-1]). These enzymes include phenylalanine ammonia-lyase, aryl aldehyde dehydrogenase, aryl-alcohol dehydrogenase, aryl-alcohol oxidase, and transaminase (aminotransferase).

Based on previous studies, the production of benzaldehydde and benzyl alcohol has been described in two pathways [58–60]. In one pathway, L-phenylalanine is initially converted into trans-cinnamic acid by the action of phenylalanine ammonia-lyase, and then cinnamic acid is converted into 3-phenylpropionic acid or benzoic acid; in the other pathway, transaminase or L-amino acid oxidase initiated the pathway leading from L-phenylalanine to 3-phenylpyruvic acid. Though the metabolic pathway in *A. blazei* is still unknown and more experiments need to be done to reveal it in *A. blazei*, identification of enzymes involved in benzaldehydde and benzyl alcohol formation will facilitate the investigation of biosynthesis pathway in this fungus.

### 3.5. Global Gene Expression Analysis and Novel Transcript Prediction

The RNA-seq Illumina Hiseq PE150 platform was employed to investigate gene expression at two key development stages, defined by mycelia (MY) and primordia (PR). Of the 10,119 predicted genes, 7,624 genes (about 75.3%) were expressed at least at one developmental stage with the cut-off FPKM value of 1. In addition, novel transcripts can be determined through high-throughput RNA-seq, which enriched the database of *A. blazei*. A total of 1,709 novel transcripts with averaged FPKM of 1 for corresponding replicates at least from one development stage were predicted at the two developmental stages mentioned above, of which 1,577 (about 92.3%) were longer than 500 bp ([Supplementary-material supplementary-material-1]).

### 3.6. Discovery of Genes Related to Fruiting Body Formation by Enrichment Analysis

**Figure 3:**
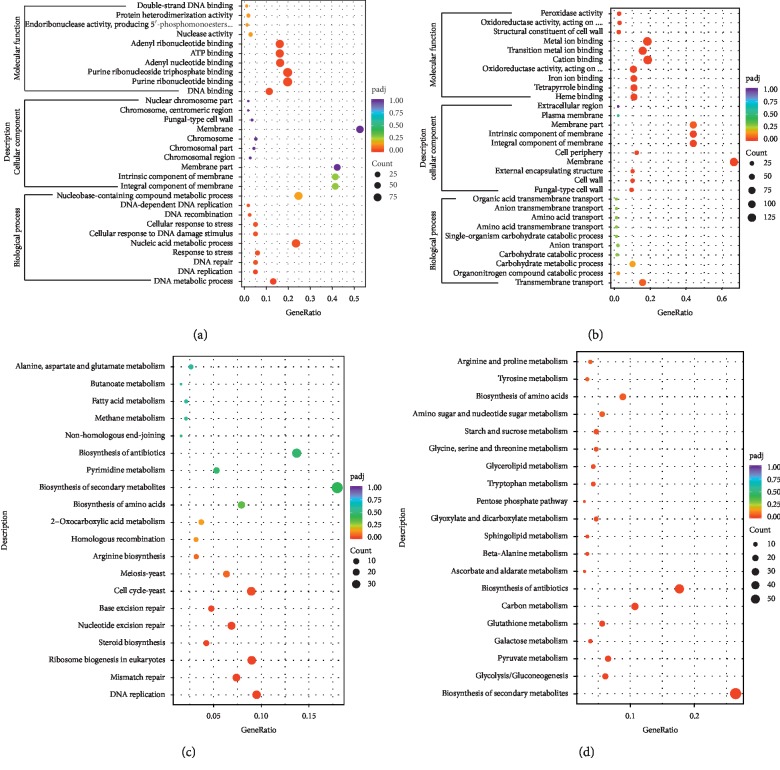
Comprehensive analysis of differentially expressed genes (DEGs) between mycelia and primordia. (a) and (b) represented GO terms enrichment analysis of upexpressed genes and downexpressed genes, respectively; Description, function description of GO term; GeneRatio, the ratio of differentially expressed genes under the corresponding GO term to the total number of differentially expressed genes; padj, adjusted *P* value; and Count, the number of differentially expressed genes under the GO term. (c) and (d) indicated KEGG pathway enrichment analysis of up- and downregulated genes, respectively; Description, function description of KEGG term; GeneRatio, the ratio of differentially expressed genes under the corresponding KEGG term to the total number of differentially expressed genes; padj, adjusted *P* value; and Count, the number of differentially expressed genes under the KEGG term.

To identify and investigate the differentially expressed genes (DGEs), the total RNA from the mycelia and primordia was used to construct DGEs library. We analyzed the DGEs and found 3,721 genes were significantly differentially expressed between mycelia and primordia, including 2,164 and 1,557 genes downregulated and upregulated in primordia, respectively. The DGEs were classified into three categories (biological process, cellular component, and molecular function) in the GO database (Figures 3(a) and 3(b)). Comparing the GO annotation of these DGEs between mycelia and primordia revealed that annotation percentage of the carbohydrate metabolic process, transmembrane transport, cell wall, integral component of membrane, peroxidase activity, antioxidant activity, O-methyltransferase activity, oxidoreductase activity, and hydrolase activity in mycelia upregulated genes were higher than those in primordia, whereas GO terms involved in response to stress, nucleic acid metabolic process, DNA replication, ribosome biogenesis, protein modification process, cytoplasm, and ATP binding showed higher levels in primordia. The formation of fruit bodies is one of the most complex processes and is affected both by external environmental factors and endogenous genes. Most of the genes involved in response to stress were upregulated in the primordia, which could improve the adaptation to environmental change during the fruiting body formation process and thus play a positive regulatory role during formation of fruit bodies.

The KEGG enrichment analysis was implemented for identification of the significantly enriched biological processes in DGEs. The result showed that several gene sets were significantly enriched (Figures 3(c) and 3(d)). Previous observations demonstrated that more genes participated in protein and energy production in mycelia of *S. commune* [44]. The DGE analysis of *A. blazei* showed that energy production such as glycolysis/gluconeogenesis, starch and sucrose metabolism, galactose metabolism, glyoxylate and dicarboxylate metabolism, pentose phosphate pathway, and pyruvate metabolism were upregulated in mycelia. Except the carbohydrate catabolic process, genes associated with arginine and proline metabolism and glycine, serine, and threonine metabolism were enriched in the group of genes downregulated in primordia. This analysis suggested that the carbohydrate catabolic process and amino acid metabolism pathways were more active in the mycelia, and the upregulation of energy production might be required for mycelia growth.

Ribosomes are ribonucleoprotein complexes found within all living cells. They have been viewed as a molecular machine that functions as the site of biological protein synthesis [63, 64]. Genes involved in ribosome biogenesis were upregulated at the primordial stage which was similar to the expression of that in *F. velutipes* [22] and *A. polytricha* [65], implying that more proteins were required during fruiting body formation. Ribosomes were also participated in DNA repair, development, and cell division [63, 66]. 18 genes involved in DNA replication were found to be upexpressed in primordia, compared with those at the mycelial stage. Genes encoding ribosome proteins and DNA replication proteins were highly expressed in primordia, which suggested that an increase in activity of ribosome and DNA replication was needed for cell differentiation during primordia formation.

Arginine, an important amino acid in plants, acts not only as the main nitrogen reserve but also as a biosynthetic precursor of polyamine, glutamic acid, and nitric oxide [67]. It is reported that arginine and its metabolites are associated with growth and development and adaptation to environmental change [67]. Besides that, arginine plays a role in accelerating and increasing fruiting body formation in fungi [68, 69]. The KEGG pathway enrichment analysis also indicated that genes under the term of arginine biosynthesis showed high level in primordia, suggesting their importance for fruiting body formation. Significant changes in the level of genes related to the arginine biosynthesis may lead to the change of arginine, which may be beneficial for *A. blaze* adapting to environment and regulation of nitrogen metabolism, thus providing sufficient nitrogen sources for fruiting body formation. Steroids are a kind of bioactive compounds in mushroom. In steroid biosynthesis, several important encoding enzyme genes showed increased expression in primordia such as squalene synthase, squalene monooxygenase, lanosterol synthase, lanosterol 14-alpha demethylase, 3-keto steroid reductase, sterol 24-C-methyltransferase, delta24 (24(1))-sterol reductase, and sterol O-acyltransferase. The upregulated expression of these genes could be conducive to accumulation of steroid in the fruiting body. Earlier investigations demonstrated that steroids provide characteristic functions that were necessary for mycelial growth and sporophore formation in mushroom [70–72]. The active steroid biosynthesis in *A. blazei* seemed to be required for fruiting body formation.

### 3.7. Genes Previously Identified as Important for Vegetative Growth and Fruiting

The DGE profiles were also employed for examining genes that have previously played a role in fungal growth and fruiting body formation [65, 73, 74]. Some genes, in accordance with the previous study, were found, namely, genes encoding metallopeptidase, glycosyl hydrolases, laccase, hydrophobin, and WD40 protein ([Supplementary-material supplementary-material-1]). Metallopeptidases have been discovered in several mushrooms such as *A. polytricha* [65] and *P. ostreatus* [73]. Higher expression of metallopeptidase in primordia and fruiting body than mycelia from *P. ostreatus* suggested that metalloprotease played an important role in the initiation and formation of fruit bodies [73]. In the present study, four metallopeptidase members were identified, and all of them displayed high expression in mycelia implying that metallopeptidase also had a critical function in the mycelial stage in *A. blazei*. Moreover, 10 glycosyl hydrolases were upregulated in primordia and 16 in mycelia demonstrating their importance for *A. blazei* growth, especially in the mycelial stage. The multigene family of laccases has been widely described in mushrooms (eg., *A. bisporus, F. velutipes*, and *V. volvacea*), and these enzymes are involved in lignin degradation [74, 75], fruiting body formation [76], and stipe elongation [19, 56]. We found that a total of 8 laccases ([Supplementary-material supplementary-material-1]) showed significant expression between the mycelial and primordial stage. Among them, 6 enzymes were upexpressed in mycelia, three of which (A04922, A05207, and A01869) exhibited more than 50-fold, 134-fold, and 1,478-fold higher expression levels than in primordia, which suggested that they possibly play important roles in lignin bioconversion in *A. blazei*. Compared with mycelia, the expression levels of A05193 and A06810 were upregulated to approximately 202 folds and 5 folds in primordia, respectively, which indicated that these two genes may be associated with fruiting body formation. These genes could play a similar function with laccase from *V. volvacea*, in which laccase could crosslink mycelial walls into coherent aggregates during the initiation of primordia and then continue to act on the mycelia surfaces throughout fruiting body development [77].

Hydrophobins, unique fungal proteins with a wide spectrum of functions, have been reported to participate with fungal growth and fruiting body formation [78–80]. *A. blazei* possessed 21 genes encoding fungal hydrophobins in mycelial and primordial stags, and the expression difference of these genes between the two stages was analyzed. Among the identified hydrophobins, A06219, A04048, A01017, and A08707 were found to have significantly upregulated expression in primordia, whereas 17 of 21 genes showed downregulated expression in primordia ([Supplementary-material supplementary-material-1]), indicating that different hydrophobins could be required for different developmental stages in *A. blazei*. WD40 repeat proteins represent a large family in eukaryotes and perform diverse functions like regulation of growth, cell cycle, development, signal transduction, and formation [81]. The upregulation of WD40 repeat protein family members indicated the abundance of these proteins in primordia.

### 3.8. Validation of the DGE Results by Quantitative Real-Time PCR (qRT-PCR)

**Figure 4:**
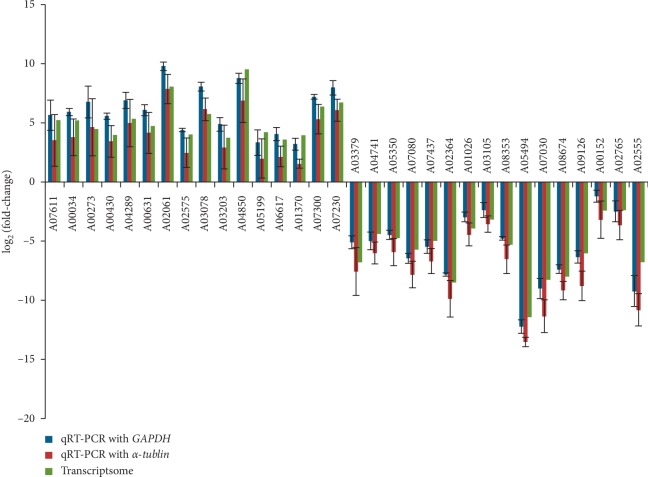
The qRT-PCR analysis of gene expression compared with the RNA-seq data in mycelia and primordia. qRT-PCR with GAPDH indicated the expression level detected using qRT-PCR with GAPDH acting as the reference gene; qRT-PCR with *α*-*tublin* meant the expression level detected using qRT-PCR with α-*tublin* acting as the reference gene.

To validate expression profiles obtained by RNA-seq, we conducted qRT-PCR for 32 randomly selected genes with *GAPDH* and *α-tubulin* acting as the reference gene (Figure 4, Tables [Supplementary-material supplementary-material-1] and [Supplementary-material supplementary-material-1]). The transcript levels of these genes analyzed by qRT-PCR were consistent with RNA-seq results (Figure 4), indicating that the DEG analysis was reliable.

## 4. Conclusion

We constructed a de novo assembly of genome sequence of *A. blazei* sterile monospore strain JA-15036 and predicted 10,119 genes from the genome sequence data. Several enzymes possibly involved in biosynthesis of *β*-1,3-glucan and bezylic compound were identified. These data will provide the basis for understanding the metabolism for benzylic compound formation in *A. blazei*. We also reported and analyzed the difference between mycelia and primordia of artificially cultivated *A. blazei* strain JA by transcriptome analysis. Functional annotations revealed that glycolysis/gluconeogenesis, starch and sucrose metabolism, galactose metabolism, glyoxylate and dicarboxylate metabolism, pentose phosphate pathway, and pyruvate metabolism were more active in mycelia while response to stress, ribosome biogenesis, arginine biosynthesis, and steroid biosynthesis pathway were more active in fruiting body. Besides, the transcript pattern of genes previously identified as important for vegetative growth and fruiting was analyzed. The expression results in our work would be useful in selecting candidate genes for further studies on the growth and development of this mushroom.

## Figures and Tables

**Table 1:** Illumina HiSeq PE150 data statistics of *A. blazei*.

Insert size (bp)	Reads length (bp)	Raw data (Mb)	Filtered reads (%)	Clean data (Mb)	Clean data Q20 (%)	Clean data Q30 (%)
350	2 × 150	1,238	8.30	1,135	96.19	90.25

**Table 2:** PacBio RSII data statistics of *A. blazei*.

Insert size (bp)	Number of reads	Number of base (bp)	Mean read length
20 kb	399,158	3,047,821,198	7,635

**Table 3:** Feature of *A. blazei* genome.

General features		Properties of predicted gene models	
Polished contigs	36	Total models	10,119
Genome size (bp)	38,686,133	Nr	8,694 (85.9%)
Gene number	10,119	Swissprot	2,102 (20.8%)
GC content (%)	49.59	Pfam	6,064 (59.9%)
Gene length	15,513,776	KOG	1,620 (16.0%)
Gene average length	1,533	KEGG	8,538 (84.4%)
% of genome (genes)	40.1	GO	6,064 (59.9%)
N50 contig length (bp)	1,826,870	CAZY	279 (2.8%)
Gene internal length	23,172,357	Total	9,174 (90.7%)
GC content in intergenic region (%)	45.35		

**Table 4:** BUSCO analysis on assembly and annotation.

BUSCO mode	Complete BUSCOs (C)	Complete and single-copy BUSCOs (S)	Complete and duplicated BUSCOs (D)	Fragmented BUSCOs (F)	Missing BUSCOs (M)	Total BUSCO groups searched
Genome	272 (93.7%)	271 (93.4%)	1 (0.3%)	6 (2.1%)	12 (4.2%)	290

## Data Availability

The genome sequences of *A. blazei* JA-15036 have been deposited at DDBJ/ENA/GenBank (http://www.ncbi.nlm.nih.gov/) under the accession number SSNC00000000, and the version described in this paper is version SSNC01000000. The transcript raw Illumina sequencing data of mycelia and primordia were submitted to NCBI Gene Expression Omnibus (GEO) with the accession numbers GSM3814130, GSM3814131, GSM3814132, GSM3814133, GSM3814134, and GSM3814135.

## Supplementary Materials

Supplementary Materials Supplementary information included 8 tables, and each of them has been named. Table S1 displayed primers used for qRT-PCR; Table S2, *Agaricus blazei* gene annotations; Table S3, carbohydrate-active enzyme (CAZy) annotation results; Table S4, the putative genes in beta-1,3 glucan and UDP-glucose biosynthesis; Table S5, the putative genes involved in biosynthesis of benzaldehyde and benzyl alcohol in *Agaricus blazei*; Table S6, list of novel transcripts identified from mycelium and primordium by RNA-Seq; Table S7, genes previously identified as important for vegetative growth and fruiting; Table S8, the expression information of selected genes for qRT-PCR in mycelium and primordium.
