# Supplementary material for: Genome Survey and Transcriptome Analysis on Mycelia and Primordia of Agaricus blazei
Source: Biomed Res Int. 2020 Jan 13;2020:1824183. doi: 10.1155/2020/1824183 (PMC6983287; doi:10.1155/2020/1824183)
Supplement: Supplementary Materials — Supplementary information included 8 tables, and each of them has been named. Table S1 displayed primers used for qRT-PCR; Table S2, Agaricus blazei gene annotations; Table S3, carbohydrate-active enzyme (CAZy) annotation results; Table S4, the putative genes in beta-1,3 glucan and UDP-glucose biosynthesis; Table S5, the putative genes involved in biosynthesis of benzaldehyde and benzyl alcohol in Agaricus blazei; Table S6, list of novel transcripts identified from mycelium and primordium by RNA-Seq; Table S7, genes previously identified as important for vegetative growth and fruiting; Table S8, the expression information of selected genes for qRT-PCR in mycelium and primordium. [file 1824183.f1.zip › supp.1824183/Figures and Supplementary Information/Supplementary Information/Table S.docx]

Table S1 Primers used for qRT-PCR

| GeneID | Primers(F, Forward; R, Reverse) |
| --- | --- |
| A07611 | F: TCATTGCGGCATTGCTTTA |
|  | R: CATTTCGGGTTGGTTGTTG |
| A00034 | F: ACGCCACCAAGGGATTTAT |
|  | R: CGATTAGTGATGGACCAAGTA |
| A00273 | F: TTCCCAGCACAGACAGCACT |
|  | R: TCGCAGCATCTCCCACCCT |
| A00430 | F: CTTCACCTCATAGCCCCGTAG |
|  | R: TGTTTCGTCAGCCTCCTTG |
| A04289 | F: GAAGGCGGGCTATTCATCAT |
|  | R: CAGAACAGGACCCAACATAACAC |
| A00631 | F: TCGGAACTGGATGGTCTTTT |
|  | R: TTACAGCGTTGGTGAGGGA |
| A02061 | F: TCAGGAAGAGGCGAAGGAGA |
|  | R: TGGTAGCGAAGCATTGGGTT |
| A02575 | F: CATAACCCGCATCGTATTGTCA |
|  | R: GATAGAAGGCTAAATGAGGTCCAA |
| A03078 | F: CTCGCAAATACGACCTTTACCC3' |
|  | R: TTCCCAATGACGGCAACC3' |
| A03203 | F: GCAACCGTCATCATCAACCA |
|  | R: TTGTCAATGGTTTCGGTGGAG |
| A04850 | F: CATTAGATGCCCAGAACGACC |
|  | R: TCTGAGTGGTTTGATTGGAGG |
| A05199 | F: ACGCCTACCCTCGTTCAGA |
|  | R: GCAAGTAAGCAACAGCACCC |
| A06617 | F: GCTTCTGGAAACCACCTTATCT |
|  | R: CACCTGCCTCTGACGAGTATCT |
| A05196 | F: AATCCCGAGTTCAGCCTTGTAG |
|  | R: CATCGTCTTGTCGCCCATCT |
| A07300 | F: ATGCGGCTGCTAATGTAGTTTC |
|  | R: GTCGTAGTGCTCGTCACCTAAATC |
| A07230 | F: CCGATAATCCAGAAGCGAAAGC |
|  | R: TGGGCAGGAAGAGGGCAAT |
| A03379 | F: CTATCTCGGAAAGACGCTGCTC |
|  | R: TGCGGGTAGTGATGATGCTGT |
| A04741 | F: CATCAAGACACTCGTGGTACTCCT |
|  | R: TCCAATCTCCTCCAACAAATCA |
| A05350 | F: TTGCCGTGACCTTCGCTAC |
|  | R: CCGTCTGACTTGGCTTTGGA |
| A07080 | R: CGGAGTGTTTAGTCGGAATAGAA |
|  | F: GAGAATTTACGGACGAGGGTC |
| A07437 | F: AAAGCGGTCGTCACTCTTCCAC |
|  | R: AAACCCGTCGTCGGTCCAT |
| A02364 | F: GCACGGTGTTCAATGTCCTC |
|  | R: ACTGCGTCGGCAAGAAATAC |
| A01026 | F: GTCGTGGCAATGGTTTCTACA |
|  | R: ACGGTCATCTGCGAGTTCTTC |
| A03105 | F: ACCCAATCGCAGCAGCAGA |
|  | R: GGGAGTAGGTGGTTGAGTGTCG |
| A08353 | F: TGAACAGCCAACCGACGAG |
|  | R: AAACGAGGACGACCCAACGA |
| A05494 | F: TCTACGAAAGAAGGGCTGGG |
|  | R: TAACATCCTGCTGAGTGCTG |
| A07030 | F: ATCCTCGCCTTAGTCATTCG |
|  | R: GCGGCTTCTTCCACTCGTT |
| A08674 | F: GTTTGGAGGGTGCTTTATCG |
|  | R: CAAGGTGACCATCGCTGTAT |
| A09126 | F: TATCAACCGTCGTATCTACTGCTC |
|  | R: GTCATATTCTTCCTCGTTTCCTGT |
| A00152 | F1:AGCCATTCATTTACCATCCATT |
|  | R1:AGTCCACCCTGATTTCCTCTTC |
| A02765 | F: GCTAGAGGAGATGCGAACAAA |
|  | R: GAGATAGTGATGGCACGAAAGA |
| A02555 | F: TGCCTTCCAGACGCCAAT |
|  | R: CTTCCCGATACGAGTCCAAAT |
| *GAPDH* | F: TTGAAGTCGTCGCTGTGAAC |
|  | R: AAGGGGCGGAGATAATGACT |
| α-tublin | F: CATTTCCCACTTGCCACTTTC |
|  | R: CAACAGCAGCATTGACATCCTTA |
